# Resistance, Resilience, and Recovery of Dryland Soil Bacterial Communities Across Multiple Disturbances

**DOI:** 10.3389/fmicb.2021.648455

**Published:** 2021-04-20

**Authors:** Blaire Steven, Michala L. Phillips, Jayne Belnap, La Verne Gallegos-Graves, Cheryl R. Kuske, Sasha C. Reed

**Affiliations:** ^1^Department of Environmental Sciences, Connecticut Agricultural Experiment Station, New Haven, CT, United States; ^2^United States Geological Survey, Southwest Biological Science Center, Moab, UT, United States; ^3^Bioscience Division, Los Alamos National Laboratory, Los Alamos, NM, United States

**Keywords:** dryland, biological soil crust, resilience, recovery, bacteria, arid soils

## Abstract

Dryland ecosystems are sensitive to perturbations and generally slow to recover post disturbance. The microorganisms residing in dryland soils are especially important as they contribute to soil structure and nutrient cycling. Disturbance can have particularly strong effects on dryland soil structure and function, yet the natural resistance and recovery of the microbial components of dryland soils has not been well documented. In this study, the recovery of surface soil bacterial communities from multiple physical and environmental disturbances is assessed. Samples were collected from three field sites in the vicinity of Moab, UT, United States, 6 to 7 years after physical and climate disturbance manipulations had been terminated, allowing for the assessment of community recovery. Additionally, samples were collected in a transect that included three habitat patches: the canopy zone soils under the dominant shrubs, the interspace soils that are colonized by biological soil crusts, and edge soils at the plot borders. Field site and habitat patch were significant factors structuring the bacterial communities, illustrating that sites and habitats harbored unique soil microbiomes. Across the different sites and disturbance treatments, there was evidence of significant bacterial community recovery, as bacterial biomass and diversity were not significantly different than control plots. There was, however, a small number of 16S rRNA gene amplicon sequence variants that distinguished particular treatments, suggesting that legacy effects of the disturbances still remained. Taken together, these data suggest that dryland bacterial communities may possess a previously unappreciated potential to recover within years of the original disturbance.

## Introduction

Soil microorganisms are central to many biogeochemical cycles, including those for carbon, nitrogen, and phosphorus. Given the significance of these populations and the processes they help control, there is a rich literature describing how microbial communities respond to various disturbances (Allison and Martiny, 2008; Shade et al., 2012; Evans and Wallenstein, 2014). The capacity of a community to recover from a disturbance is referred to as the community’s resilience (Baho et al., 2012). Thus, a community’s resilience defines the severity of population shifts in response to a disturbance and the time scales at which recovery can be expected to occur. This, in turn, informs the scale and resources that should be dedicated to management or intervention efforts to restore degraded ecosystems (van de Leemput et al., 2018). Information on the resilience of soil microbial communities is severely lacking (García-García et al., 2019; Rath et al., 2019; Uritskiy et al., 2019). A recent meta-analysis of microbial disturbance and recovery found that only a fraction of studies undertake assessing the recovery of microbial communities as an objective, and only a small number of those studies measure or detect a return to pre-disturbance community composition and function. Thus, the authors posit that this either reflects that microbial communities have not been sampled with enough replication or duration to assess recovery, or that microbial populations in fact have a low resilience to disturbance (Shade et al., 2012). Consequently, there is considerable uncertainty regarding the resilience of microbial communities to the multitude of disturbances they face in a changing environment and under altered land use.

Drylands account for approximately 40% of the terrestrial surface, and are expected to expand under a regime of climate warming (Cherlet et al., 2018). Arid ecosystems are among the most fragile areas on the planet, and thought to be exquisitely sensitive to perturbations due to their low and limited resource availability (Archer and Predick, 2008; Poulter et al., 2014). This resource scarcity leads to generally slow recovery rates of arid ecosystems post disturbance. For example, estimates for plant recovery in arid ecosystems ranges from 60 to >100 years depending on factors such as elevation, temperature, and rainfall (Estruch et al., 2018; Monroe et al., 2020). Similar recovery times have been proposed for surface soil autotroph communities (i.e., biological soil crusts) with full recovery proposed to occur in a decadal or even century timeframes (Weber et al., 2016; Williams et al., 2018). However, these studies have often focused on late successional dryland soil communities such as mosses and lichens with relatively little information on the pioneering soil bacterial communities.

In 2015, we reported the response of dryland soil bacteria to multiple climate change and physical disturbance manipulations (Steven et al., 2015). These consisted of chronic physical trampling, a 4∘C above ambient warming, altered precipitation consisting of increased frequency of small precipitation events, and the warming and precipitation manipulation in combination. The disturbances generally resulted in significant declines in bacterial biomass and shifts in the relative abundance of hundreds of bacterial taxa (Steven et al., 2015). In several cases the disturbances resulted in a total collapse of the surface biological soil crust communities, which are composed of cyanobacteria, algae, lichens, mosses and their associated heterotrophic fungi, bacteria, and archaea (Housman et al., 2007; Steven et al., 2012a, 2014; Steven, 2017). Yet, we found that each disturbance resulted in a unique microbial community composition, which was not apparent at the visual-scale (Ferrenberg et al., 2015). Given the severity and differing responses of the communities to the perturbations, we undertook a study to characterize the recovery of the bacterial populations following the termination of these disturbances. Chronic trampling and the precipitation pulse disturbances were terminated 6–7 years prior to sampling, while the warming treatment was maintained. In this manner, we could assess if increased temperatures would influence the rate or success of recovery of the soil bacterial communities. We further investigated three habitat patches: (1) root zone soils under the canopy of the local shrubs, (2) interspace soils colonized by biological soil crusts, and (3) the edge soils at the border of the plot. With this approach, we could test if recovery was happening equally across the plot or if particular environmental niches fostered a faster recovery.

## Materials and Methods

### Sample Sites and Field Manipulations

Three long-term *in situ* experiments located in otherwise undisturbed natural dryland landscapes were used for this study. The effects of chronic physical disturbance by annual foot trampling were tested at two sites, one located in Arches National Park (termed Arches here; 38.72662 N, 109.5434 W, elevation 1,490 m). At Arches, soil depth ranged from 30 to 100 cm and was of the Mido-Sazi complex, gray soil, loamy sand. The dominant shrub was *Coleogyne ramosissima* (blackbrush).

The second trampling site was located in the Island in the Sky district of Canyonlands National Park (termed ISKY here; 38.45756 N, 109.5411 W, elevation 1,800 m). The ISKY soil profile was 10–25 cm in depth and consisted of Rizno dry rock outcrop complex, light blond soil, loamy sand. The dominant shrub was also *C. ramosissima* (blackbrush). Both trampled sites harbored biological soil crusts that differed in development and composition of lichens and cyanobacteria, where cyanobacteria were more abundant and developed at the ISKY site.

The third site, Castle Valley, UT, United States (termed CV here; 38.6748 N, 109.4163 W, elevation 1,310 m), was about 10 km from the Arches and ISKY sites. Soil depth ranged from 17 to 122 cm and was a Lithic Torriorthent, a sandy loam. The dominant shrub was *Atriplex confertifolia*. Here, the effects of year-round warming and altered frequency of summer precipitation were tested in a long-term field experiment. Increased warming with infrared (IR) lamps was delivered to five replicate plots, resulting in an approximately 4∘C soil warming to a depth of 5 cm. Altered precipitation was delivered as 1.2-mm water pulses in an average of 35 events during the summer months, resulting in an approximate 4-fold increase in the number of small precipitation events. Separate plots received year-round warming and altered precipitation treatments in combination. Control plots consisted of untreated plots with a non-energized IR lamp housing. Plots were 2 m × 5 m in size with five replicates per treatment per site in a randomly assigned full factorial design.

Across the three field sites, annual precipitation from 2005 to 2018 ranged from 137 to 375 mm with an average of 246 mm. The manipulations employed in these studies were within the range of climate or land use changes already occurring in these regions or predicted to occur within the next few decades (Wang J. et al., 2007). Samples were collected from Arches and from ISKY on October 1st, 2018. Both sites received a cumulative 16 years of annual spring trampling treatments. At the time of sampling the treatments had been ended for 7 years. At the CV site, samples were collected on October 2nd after 6 years of treatment. All treatments, excluding warming, were terminated in 2012.

### Soil Collection for Microbial Community Analysis

**FIGURE 1:**
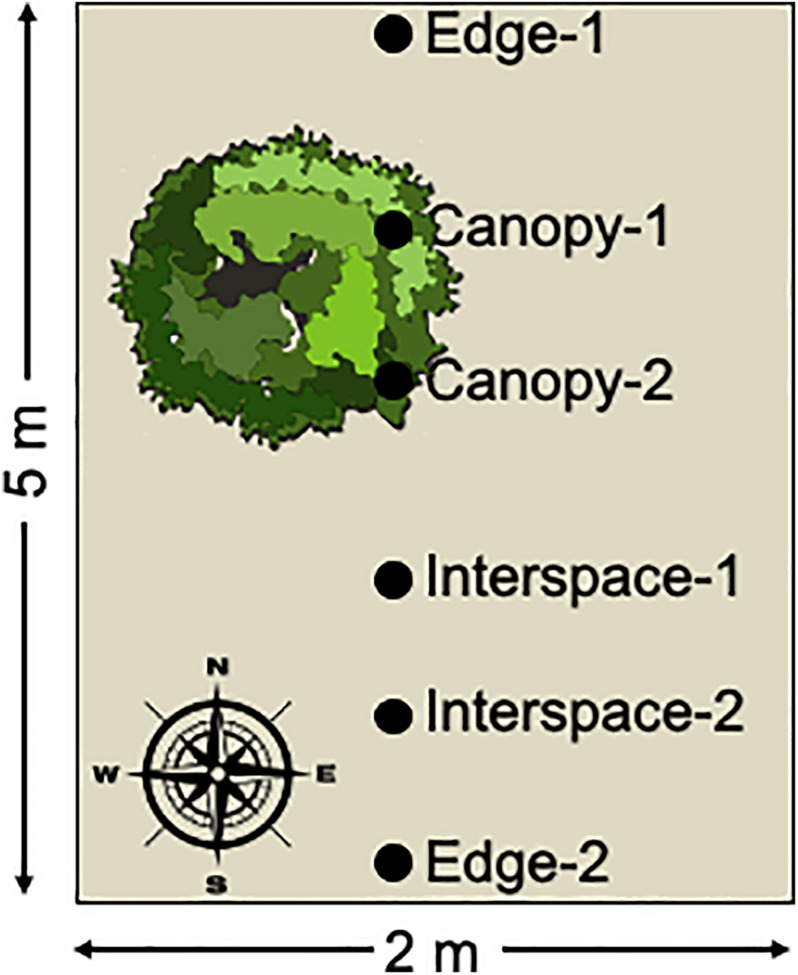
Schematic diagram of sample collection. Surface soil samples were collected along a roughly north to south transect from each sampled plot. The transect encompassed three habitat patches: canopy zone soils in the dripline of the dominant shrubs, interspace soils in the unvegetated patches of the plot, and edge soils at the plot boundaries. Two replicate samples were collected for each habitat patch and four plots were sampled per treatment, for a total of 8 biological replicates per site, treatments, and habitat patch, resulting in a total of 192 soil samples collected.

Soil samples consisted of the upper 0–2 cm of soil collected with a sterile spatula. Soil samples were immediately placed on dry-ice and were kept frozen during transport to the Los Alamos National Laboratory. The samples were stored in a −80∘C freezer until sample processing. The soil samples were collected in a roughly north to south transect from each plot encompassing the three habitat patches: the edge of the plot, canopy zone soils, and interspace soils, with two samples per habitat (Figure 1). Four replicate plots were sampled per treatment resulting in 8 replicate soil samples per habitat zone per treatment.

### DNA Extraction and Quantitative PCR

Total DNA was extracted with the Qiagen DNeasy PowerSoil HTP 96 Kit following the manufacturer’s protocols. Prior to amplification, DNA samples were normalized to a concentration of 1 ng μl^–1^ as measured by a Qubit Fluorometer. Reaction volumes of 24 μl were prepared as follows: 12.5 μl iQ Syber Green Super Mix, 0.25 μl BSA, 0.7 μl forward primer (338-Eub 5′CTC CTA CGG GAG GCA GCA CT 3′), 0.7 μl reverse primer (513-Eub 5′ATT ACC GCG GCT GCT GG 3′), 9.85 μl H_2_O. Thermal cycling consisted of initial denaturation at 95∘C for 3 min, followed by 40 cycles of 95∘C for 15 s; 55∘C for 30 s (data collection step); 72∘C for 30 s. A melting curve was performed consisting of 80 cycles at 55∘C for 10 s. A six-point standard curve was prepared using a 10X dilution series of *Microcoleus vaginatus* DNA.

### 16S rRNA Gene Amplification and Sequencing

DNA extractions were processed at the University of Connecticut’s Microbial Analysis, Resources, and Services facility for 16S rRNA gene amplifications and sequencing. Bacterial 16S rRNA genes were amplified using 30 ng of extracted DNA from each of the 192 soil samples. The V4 region was amplified using primers 515F (GTGYCAGCMGCCGCGGTAA) and 806R (GGACTACNVGGGTWTCTAAT) with Illumina adapters and dual indices. Samples were amplified in triplicate using GoTaq (Promega) with the addition of 10 μg BSA (New England BioLabs), and the three independent PCR reactions were pooled prior to sequencing. The PCR reaction was incubated at 95∘C for 3.5 min, then 30 cycles of 30 s at 95.0∘C, 30 s at 50.0∘C and 90 s at 72.0∘C, followed by final extension as 72.0∘C for 10 min. PCR products were pooled for quantification and visualization using the QIAxcel DNA Fast Analysis (Qiagen). PCR products were normalized based on the concentration of DNA then pooled using the QIAgility liquid handling robot. The pooled PCR products were cleaned using the Mag-Bind RxnPure Plus (Omega Bio-tek) according to the manufacturer’s protocol. The cleaned pool was sequenced on the MiSeq platform using v2 2 × 250 chemistry (Illumina, Inc).

### Bioinformatic and Statistical Analyses

Sequencing reads were assembled into contigs and quality screened by using mothur v1.39.5 (Schloss et al., 2009). Sequences that were at least 253 bp in length, contained no ambiguous bases, and no homopolymers of more than 8 bp were used in the analysis. Chimeric sequences were identified by using the VSEARCH (v2.15.2) as implemented in mothur (Rognes et al., 2016), and all potentially chimeric sequences were removed. To maintain a similar sampling effort between samples, datasets with less than 1,000 sequences per sample were also removed. After removal a total of 172 samples remained from the original 192 samples (Figure 1). Thus, the majority of samples contained 8 biological replicates across site, treatment and habitat patch, with the smallest number of replicates being 6. The resulting list of unique sequences was considered the set of amplicon sequence variants (ASVs). Taxonomic classification of sequences was performed with the Ribosomal Database Project classifier against the SILVA v132 reference alignment in mothur (Wang Q. et al., 2007; Quast et al., 2012).

Sequence data were imported into the phylsoseq package in R (v. 1.34.0) for exploratory and statistical analyses (McMurdie and Holmes, 2013). For calculation of Shannon’s diversity, data were randomly subsetted to the size of the smallest dataset (1,417 sequences). The subsetted data were used to calculate Bray-Curtis similarities for sample distances. Statistically significant differences in clustering were determined by permutational multivariate analysis of variance (PERMANOVA) calculated with the adonis function in the VEGAN R software package (Dixon and Palmer, 2003, v. 2.5–7).

Differentially Abundant Amplicon Sequence Variants (DA ASVs) were determined using unnormalized ASV counts with the ALDEx2 (v. 1.18.0) software package (Fernandes et al., 2013), after ASVs consisting of five or fewer sequences were removed. Briefly, a Dirichlet-multinomial model to infer abundance from counts was employed. For three-way comparisons among the field sites significant differences were identified with the Kruskal-Wallace and generalized linear model tests with raw *P*-values corrected with Benjamini-Hochberg correction. Significant ASVs had corrected *P*-values < 0.05 for both tests. For two-way comparisons among treatments the results of the Wilcoxon Rank Sum were used to identify DA ASVs. *P*-values were corrected with the Benjamini-Hochberg correction for multiple testing. Reported DA ASVs had a corrected *P*-value < 0.05. Statistically significant differences in phylum level taxonomic bins were identified using the STAMP software package (v.2.1.3) using an ANOVA test followed by a Games-Howell *post hoc* test. *P*-values were corrected for multiple testing employing Storey’s FDR method (Parks et al., 2014).

## Results

### Bacterial Community Composition, Diversity, and Biomass

**FIGURE 2:**
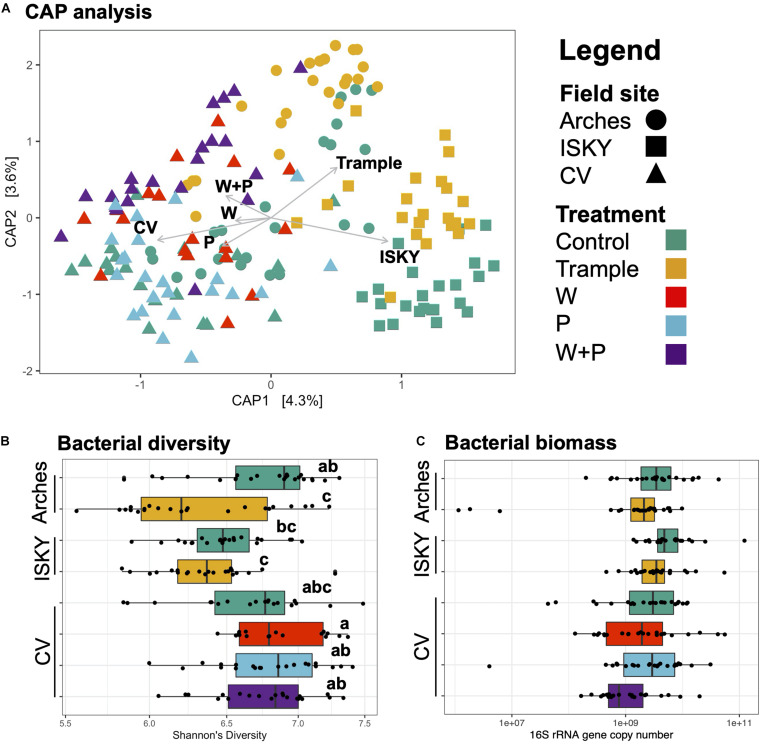
Bacterial composition, diversity, and biomass across the datasets. **(A)** Canonical analysis of principal coordinates ordination of sequence datasets. Axes labels denote the percent variation explained by principal components. Arrows indicate the direction and magnitude of the variance explained by field site and manipulation treatments. **(B)** Bacterial diversity. Diversity was assessed by the non-parametric Shannon’s diversity index. Boxplots show the mean (solid bar) and interquartile range of the values. Each individual sample is represented by the black points. Samples labeled with different letters were significantly different by ANOVA testing of means followed by a *post hoc* Tukey-Kramer test. **(C)** Bacterial abundance as determined by qPCR of bacterial 16S rRNA genes. Boxplots show the mean (solid bar) and interquartile range of the values. Each individual sample is represented by the black points. No significant differences between datasets were identified.

Canonical analysis of principal coordinates (CAP) analysis was employed to investigate the relationship between sequence datasets (Figure 2A). The largest factor explaining the variation between datasets was field site, with the samples from the three sites clustering independently (Adonis *R*^2^ 0.067, *P* < 0.001). The different treatments also clustered independently (Adonis *R*^2^ 0.071, *P* < 0.001). For example, the Arches and ISKY trampled datasets clustered distinctly, indicating that the field site differences were still apparent even in the disturbed soils (Figure 2A). Similar dynamics are observed for the CV field site, with clear differences between the warming (W), precipitation (P), and warming and precipitation (W+P) datasets (Figure 2).

To test if there were differences in bacterial diversity among the sites Shannon’s diversity index was calculated (Figure 2B). There was a significant reduction in diversity between the control and trampled datasets at the Arches field site (*P* < 0.001). Similarly, there was a reduction in diversity at the ISKY site with trampling, although the difference from the ISKY controls was not significant. Across the CV manipulations there was a general trend in diversity increasing with the manipulations, although again the differences were not significant. Taken together, these observations suggest that bacterial diversity was generally similar between the control and disturbed soils, with the exception of the trampling manipulation, particularly at the Arches site. Finally, we employed quantitative PCR (qPCR) as a measure of bacterial biomass in the soil samples (Figure 2C). Across the experiment there were no significant differences in 16S rRNA gene copy numbers between field sites or manipulations, indicating that bacterial biomass was similar across filed site and manipulations.

### Biogeography of Soil Communities Among Field Sites

**FIGURE 3:**
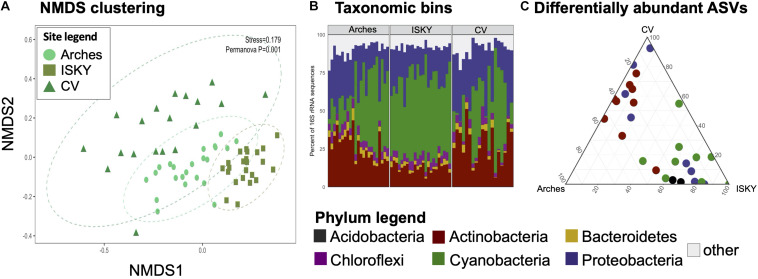
Field site biogeography. For each dataset the control samples were compared to investigate if the normal state of the soil microbiome differed among sites. **(A)** NMDS clustering. The stress value of the ordination is indicated. Ellipses indicate the 95% confidence interval of the group centroids. PERMANOVA statistical testing identified field site as a significant factor in discriminating the bacterial communities (*P* = 0.001). **(B)** Phylum level taxonomic bins in the datasets. Stacked bar graphs representing the relative abundance of the six most predominant bacterial phyla in the datasets. Each phylum accounted for at least 1% of the recovered sequences across the individual samples. The remaining taxa were assigned to the category “other.” Four phyla were identified as significantly different in relative abundance between sites, Actinobacteria, Bacteroidetes, Cyanobacteria, and Proteobacteria. **(C)** Ternary diagram of differentially abundant ASVs. Each point represents an ASV identified as significantly different in relative abundance among the sites. Points are colored by the Phylum to which they belong. A full list of the differentially abundant ASVs are listed in Supplementary Table 1.

Given that field site was found to be a large factor in structuring the bacterial communities (Figure 2A), differences in bacterial populations among field sites were investigated. The control sequence datasets were bioinformatically separated and compared by non-metric multidimensional scaling (NMDS; Figure 3A). Clustering of the datasets was readily apparent and significant (*P* < 0.001), indicating that the natural state of the soil microbiome differs among the field sites.

To characterize the populations that account for field site differences the sequences were classified to the phylum level (Figure 3B). The relative abundance of Cyanobacteria was higher at ISKY than at Arches or CV (*P* < 0.001 and *P* < 0.01, respectively). This translated into significantly lower relative abundances of the Actinobacteria at ISKY in comparison to the other field sites.

Finally, the data were interrogated for differentially abundant (DA) 16S rRNA gene ASVs. A total of 28 ASVs were identified as being significantly different in relative abundance among the sites (Figure 3C). The DA ASVs belonged to four bacterial phyla the Acidobacteria, Actinobacteria, Proteobacteria, and Cyanobacteria. Patterns in the identified ASVs matched observations for the phylum level bins. For instance, all of the ASVs related to the Cyanobacteria were enriched at ISKY, which also showed the highest proportions of cyanobacteria-related sequences (Figure 3B). Several of the DA ASVs were classified to similar taxonomic ranks. Of the 9 DA ESVs classified to the Actinobacteria, six were identified as belonging to the family *Geodermatophilaceae*. Similarly, among the eight DA ASVs related to the Proteobacteria, three belonged to the same genus, *Microvirga* (Supplementary Table 1). Thus, there was a conserved taxonomic signal among those ASVs that differed in relative abundance among field sites.

### Differentially Abundant Amplicon Sequence Variants in Response to Disturbance

**FIGURE 4:**
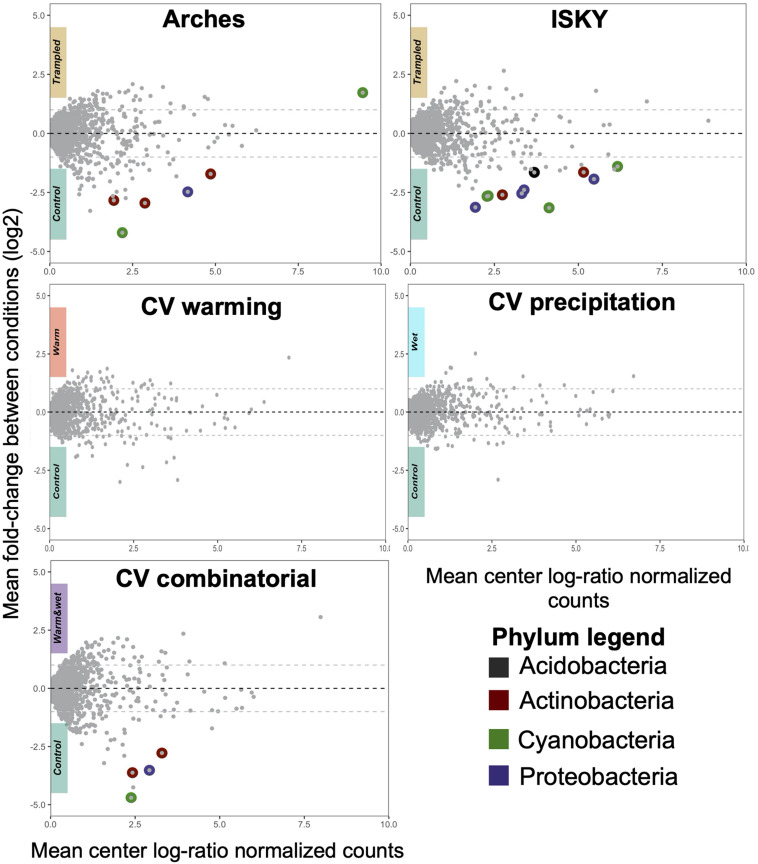
Differentially abundant ASVs due to treatment. Each panel represents a Bland-Altman plot for the abundance and fold change of each ASV in the dataset. The *x*-axis indicates the center log-ratio normalized counts of each ASV in the data, while the *y*-axis shows the fold-change in relative abundance due to the treatments. ASVs that showed a statistically significant difference in relative abundance are highlighted by colors representing the Phylum to which they belong. A full list of the differentially abundant ASVs is listed in Supplementary Table 2.

To assess the recovery of the soil bacterial communities from the different manipulations, DA ASVs between the control and treatment datasets were determined. The trampling treatments demonstrated a small number of DA ASVs with 6 and 11 in the Arches and ISKY sites, respectively (Figure 4). At both sites the majority of the ASVs were enriched in the control plots. In fact only a single ASV, classified to the cyanobacterial genus *Tychonema*, was found to be more abundant in the trampled plots at the Arches site. Interestingly four DA ASVs were identified in both the Arches and ISKY field sites (Supplementary Table 2). These ASVs were identified to the genera *Solirubrobacter* (phylum Actinobacteria), *Geodermatophilus* (phylum Actinobacteria), *Rubellimicrobium* (phylum Proteobacteria), and *Mastigocladopsis* (phylum Cyanobacteria). Thus, there appears to be common core of bacteria that show a similar response to trampling between the two field sites. A full list of DA ASVs is shown in Supplementary Table 2.

At the CV field climate change manipulation experiment, there were no DA ASVs associated with the warming (W) or altered precipitation (P) manipulations, suggesting that the soil bacterial communities have largely recovered or were resilient to the disturbances (Figure 4). In the combined treatment (W + P) a small number of ASVs were depleted relative to the control plots (Figure 4), suggesting there were still legacy effects of the W + P treatment 6 years after the altered precipitation ceased. Of note is that two of the DA ASVs were also common to the trampling manipulations. These ASVs were classified to the genus *Rubellimicrobium* (phylum Proteobacteria) and family *Micromonosporaceae* (phylum Actinobacteria), further supporting that particular bacterial taxa are consistently identified as associated with legacy effects of disturbance.

### Influence of Habitat Patches on Community Composition

**FIGURE 5:**
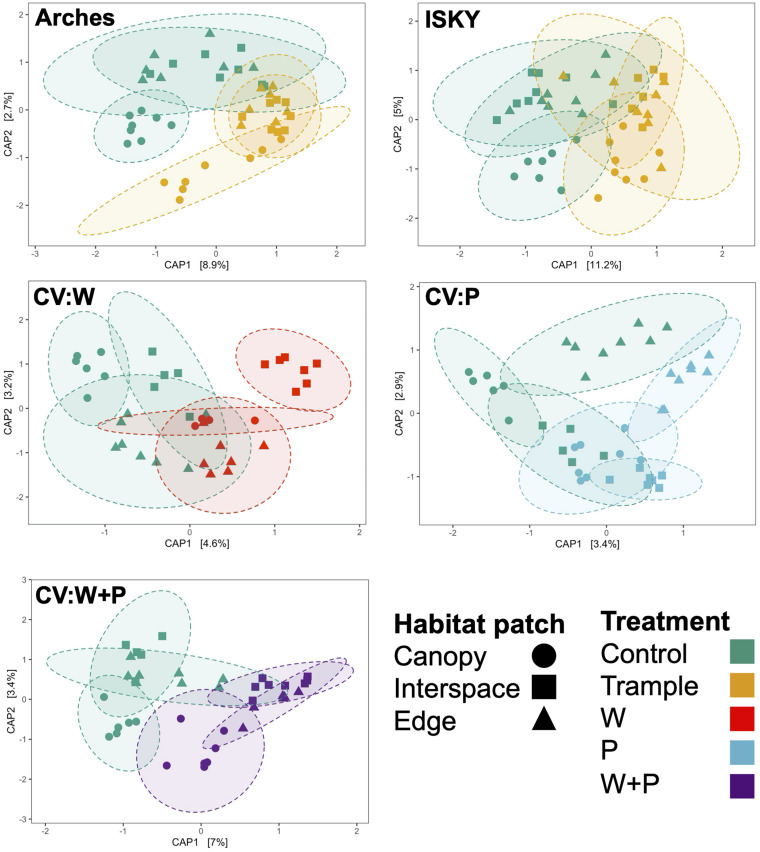
Influence of habitat patch on community composition. Canonical analysis of principal coordinates ordination of sequence datasets for each treatment. Axes labels denote the percent variation explained by principal components. Ellipses indicate the 95% confidence interval of the group centroids. A table of PERMANOVA statistical results of between group comparisons is presented in Supplementary Table 3.

Finally, we assessed three different habitat patches -canopy zone soils, interspace soils, and soils from the edge of the plot – in an attempt to test if habitat niches affect community composition or recovery. CAP clustering was employed to investigate community similarity amongst the habitat patches under the different treatments. Across the datasets the control canopy zone soils clustered independently from interspace and edge soils (*P* ≤ 0.01; Supplementary Table 3), indicating significant differences in community composition between the environments. There was a more pronounced overlap between the interspace and edge samples (Figure 5 and Supplementary Table 3). This is somewhat expected as the edge samples were interspace soils that happened to occur at the plot borders, thus it is not surprising that they represent a similar environment. Some site specific patterns were apparent. For example, in the warming plots the edge samples clustered distinctly and significantly from the other soil patches (Figure 5, *P* < 0.05; Supplementary Table 3). This may represent an experimental artifact, as the IR lamp is over the center of the plot, thus warming may have attenuated at the plot edges resulting in an unequal disturbance across the plot. In this regard, the plot interspaces potentially receive more IR warming manifesting as a larger community shift. Yet, in general, the different habitat patches shifted in the same direction and with relatively similar magnitude, suggesting that the different types of disturbance affected the soil communities to a similar degree. Thus, these data indicate that while there are differences in soil microbial populations between the habitat patches, these populations show a similar sensitivity, response, and recovery from the various manipulations performed in this study. These data also suggest that recovery was similar across the niches in the plot. For instance, the edges did not show a higher recovery than the interspaces, suggesting that recovery did not occur through migration of healthy populations from adjacent healthy soils.

## Discussion

### Biogeographic Patterns in Soil Communities

To properly assess the resilience and recovery of microbial communities, the natural state of the community first needed to be defined. This included the biogeographic factors that can influence community composition. Previous work has shown that soil edaphic factors, climatic legacies, and various landscape features all act to influence the structure of dryland soil microbial populations (Briggs and Morgan, 2008; Steven et al., 2013; Gombeer et al., 2015; Eldridge and Delgado-Baquerizo, 2019). In line with these observations, we show the bacterial populations were largely differentiated among field sites in the absence of disturbance (Figures 2A, 3). One of the ways in which the community differences were manifest was an increase in spatial heterogeneity at particular sites. For instance, the inter-sample distances between the CV replicate datasets was larger than for ISKY, with Arches falling between the two (Figure 3A). This is also apparent for the taxonomic bins identified in the datasets. The proportion of Cyanobacteria-related sequences varied widely between CV samples, while remaining more stable at the ISKY sites (Figure 3B). These observations support and build substantially on previous reports describing the biogeographic patterns that shape dryland surface soil bacterial communities (Steven et al., 2013; Wang et al., 2017; Ji et al., 2020). These data will improve our understanding of how factors such as climate and soil type impact dryland soil bacterial community composition and will inform forecasts of how these microbial populations may respond to the variety of disturbances they face in a changing future.

A second biogeographic pattern related to the different habitat patches was also apparent. In general, communities from the canopy zone and interspace soils clustered distinctly from one another, indicating differing composition (Figure 5 and Supplementary Table 3). This matches previous observations supporting that habitat patches in arid landscapes harbor differing resources and microbial populations (Schade and Hobbie, 2005; Koyama et al., 2018). Specifically, shrubs in arid ecosystems foster a collection of resources through intercepting wind carried dust, depositing carbon and nutrients to soil through litter, concentrating precipitation, and providing shade which reduces evaporation and protects from ultraviolet radiation (Coppinger et al., 1991; Martinez-Meza and Whitford, 1996; Dean et al., 1999). This generally results in an increase in microbial activity in the canopy zone soils (Conant et al., 1998; Su et al., 2004). As such, canopy zones have been referred to as “islands of fertility” (Schlesinger et al., 1990). Yet, although the composition of soil communities differed, canopy zone and interspace soils have been found to harbor an overlapping set of bacteria, suggesting that different habitat patches largely consist of a common core of species that differ in their relative abundance rather than presence or absence (Steven et al., 2012a, 2014). A similar pattern is noted here. For instance, several ASVs were identified that differentiated the field sites (Figure 3). Yet, these ASVs differed in relative abundance not presence or absence. Thus, these data suggest that a population of bacterial species is relatively ubiquitous across the landscape, but these populations shift in abundance in relation to biographic factors related to soil edaphic properties, local climates, and/or small-scale environmental niches. These are all important factors when considering the resilience of microbial communities as the different starting points for each community will likely influence how these communities respond to and recover from various disturbances.

### Recovery From Chronic Trampling

Chronic physical trampling was carried out at two field sites for 15 years. Samples collected during this disturbance showed a collapse of the soil microbial community, with reduced bacterial biomass and shifts in the abundance of dominant bacterial populations (Kuske et al., 2012; Ferrenberg et al., 2015; Steven et al., 2015, 2018). In 2015, we reported a significant decrease (approximately 2 fold lower) in 16S rRNA gene copy number in response to trampling before recovery was initiated (Steven et al., 2015). In this study, 7 years after the trampling ceased, this biomass difference was no longer apparent (Figure 2C). Yet, there were still significant differences in bacterial diversity (Figure 2B), clustering of sequence datasets (Figure 2A), and a number of DA ASVs that distinguished the control and trampled datasets (Figure 4). Therefore, while bacterial biomass may be showing signs of recovery, community composition still reflects legacy effects of the trampling disturbance. An interesting observation from the community differences due trampling was that despite biogeographic differences between the Arches and ISKY field sites (Figure 2), an overlapping set of four ASVs, all enriched in the control sites, were identified as DA at both sites (Supplementary Table 2), indicating a congruent response to chronic physical disturbance. These observations suggest that certain taxa are slower to recover and may be indicative of legacy effects of a severe disturbance.

### Response to Chronic Warming

The 4∘C warming was the only manipulation that was still in operation at the time of sampling. Yet, bacterial 16S rRNA gene copies (Figure 2C), bacterial diversity (Figure 2B), and ASV relative abundance (Figure 4) were all similar between control and warmed plots. In 2015, we reported a limited influence of warming on the soil bacterial community. There was no significant difference in bacterial biomass between warmed and control plots, and only a small number of taxa (5 genera) showed a significant difference in relative abundance (Steven et al., 2015). This points to these soil bacterial populations being largely resistant to warming above ambient temperatures. It is important to note that the visible (non-microscopic) biological soil crust community of mosses and lichens did respond to this warming treatment (Ferrenberg et al., 2015), suggesting that various members of dryland soil communities may display differing capacities to resist disturbance.

Larger magnitude warming regimes, in the scale of 13∘C, have been shown to induce a shift in the dominant bacterial populations in these systems, namely an ecological replacement of the dominant cyanobacterial population (Garcia-Pichel et al., 2013). However, these temperature shifts are significantly higher than those used in our studies, or those likely to occur with near-term climate change. This suggests that these dryland soil bacterial populations are largely resilient to the temperature increases expected to occur with near future climate warming. While changes in fungi and archaea could follow distinct patterns, these results add to a growing list of disturbances for which bacterial communities show resistance, including elevated atmospheric CO_2_ and nitrogen deposition (Steven et al., 2012b; Mueller et al., 2015). Taken together, these observations indicate that despite the well-documented sensitivity of arid ecosystems to disturbance, there are particular perturbations to which the surface soil microbial communities are resistant.

### Recovery From Altered Precipitation and Altered Precipitation and Warming in Combination

The precipitation treatment resulted in an increase in the overall amount of applied water but delivered in numerous small pulses. After 2 years of this treatment there was a significant decline in biological soil crust cover and bacterial biomass, indicating a detrimental effect on the soil populations (Ferrenberg et al., 2015; Steven et al., 2018). It was hypothesized that the declines in biomass and shifts in community composition were driven by carbon starvation induced by an increase in the number of precipitation events that do not meet a threshold to support viable photosynthesis (Coe et al., 2012). In this model, a precipitation event is associated with a rapid burst of respiration, followed by photosynthesis and carbon fixation. However, the community needs to stay hydrated long enough for photosynthesis to recuperate carbon loss induced during respiration. If the precipitation events are particularly small or of short duration a negative carbon balance can occur, resulting in carbon starvation and cell death (Reed et al., 2012). The additive effects of these small precipitation pulses delivered over multiple years leads to shifts in the composition and biomass of the soil community (Ferrenberg et al., 2015; Steven et al., 2015). Here we show that 6 years after the precipitation treatments have ceased, bacterial biomass (Figure 2C), bacterial diversity (Figure 2B), and microbial community composition (Figure 4), have largely recuperated. This suggests that the biological soil crusts exposed to an altered precipitation regime are on a trajectory to full recovery.

When the warming and precipitation treatments were combined the collapse of the soil communities was catastrophic. After 2 years of the combinatorial treatment, healthy late successional biological soil crusts were eliminated in the interspace soils. This was verified through significant reductions in cyanobacterial biomass, and a dysbiosis in the soil microbial community with hundreds of taxa displaying significant shifts in relative abundance in comparison to control plots (Steven et al., 2015). A 1° temperature warming can increase soil evaporation by 3–5% (Le Houérou, 1996). By combining a 4∘C warming with frequent small precipitation events, the number of deleterious precipitation events may be greatly increased, producing detrimental results for the soil communities. When the precipitation treatment was removed (leaving the warming in place), the soil communities showed signs of recovering. Bacterial biomass (Figure 2C) and composition (Figure 3) were similar to control plots, although some legacy effects are apparent. Four ASVs were still significantly different between the W + P and control plots (Figure 4 and Supplementary Table 2). Collectively, these data show that the interactions among changing temperature and precipitation regimes that may occur in this region have the potential to influence the soil microbial communities, and further demonstrate how environmental disturbances can act in concert to drive microbial communities to states that would not be predicted from investigating individual disturbances in isolation. Finally, we also show that when one of the disturbances is removed the community retains the capacity for recovery.

## Conclusion

The observations of this study support the idea that these dryland soil bacterial communities show a significant resistance and resilience to a number of disturbances, in that they either were recalcitrant to change in the face of the disturbance (i.e., warming) or have begun to show a process of recovery when the disturbances end. Interestingly, this was true for two very different forms of disturbance: physical trampling and altered precipitation. Thus, the natural recovery of dryland soil bacterial communities may be occurring at previously unrecognized scales. Instead of decades to centuries we are witnessing significant recovery in just 6–7 years. However, these results need to be treated with caution. The data presented here suggest that the taxonomic composition of the communities is recovering but does not address their functional attributes. Recently it was shown that disturbed arid soil communities differed significantly in their transcriptional response to a wetting pulse in comparison to undisturbed control soils, despite harboring a similar community composition (Steven et al., 2018). For instance, in control surface soils thousands of transcripts, related to processes such as photosynthesis and the Calvin-Benson Cycle were differentially expressed due to a short 1-h wetting pulse. In contrast, trampled soils showed relatively few differentially expressed genes, suggesting a muted response to wetting (Steven et al., 2018). Thus, the metabolic capacity of disturbed soil communities may differ even when the community composition is similar.

These arid soil communities provide a wealth of ecosystem services to drylands, including soil stabilization, nitrogen fixation, and moisture retention among others (Neilson et al., 2017; Bowker et al., 2018; Feng et al., 2020). Measuring the true recovery of these communities will require addressing if they are functionally, as well as taxonomically, equivalent to undisturbed populations. Finally, this study only addresses the bacterial component of the community. Other members such as fungi, lichens, and mosses also play a significant role in dryland ecology (Xiao and Veste, 2017; Green et al., 2018), but were not addressed in this study. Taken together, these data all point to significant knowledge gaps in the natural recovery rate and resilience of dryland soil populations. In particular, the data presented here suggest the sensitivity and recovery of these communities has been largely underestimated, and more data are needed to determine the consistency and drivers of recovery patterns across drylands. Through a long-term program tracking of the recovery of these systems to a multitude of disturbances we can better inform management and restoration efforts in order to protect these important ecosystems.

## Data Availability Statement

The datasets presented in this study can be found in online repositories. The names of the repository/repositories and accession number(s) can be found below: https://www.ncbi.nlm.nih.gov/genbank/, PRJNA682175.

## Author Contributions

BS, JB, CK, and SR contributed to study design, data analysis, and manuscript writing. MP contributed to experimental methods, data interpretation, and manuscript editing. LG-G contributed to experimental methods and data analysis. All authors contributed to the article and approved the submitted version.

## Conflict of Interest

The authors declare that the research was conducted in the absence of any commercial or financial relationships that could be construed as a potential conflict of interest.
